# Topical efinaconazole: A sequential combination therapy with oral terbinafine for refractory tinea unguium

**DOI:** 10.1111/1346-8138.15973

**Published:** 2021-05-24

**Authors:** Hiromitsu Noguchi, Masahide Kubo, Kayo Kashiwada‐Nakamura, Katsunari Makino, Jun Aoi, Satoshi Fukushima

**Affiliations:** ^1^ Noguchi Dermatology Clinic Kumamoto Japan; ^2^ Department of Dermatology Japan Community Health Care Organization Kumamoto General Hospital Kumamoto Japan; ^3^ Department of Dermatology and Plastic Surgery Faculty of Life Sciences Kumamoto University Kumamoto Japan

**Keywords:** combination therapy, efinaconazole, onychomycosis, terbinafine, tinea unguium

## Abstract

Efinaconazole is a topical antifungal drug approved in Japan for tinea unguium. Although topical treatments generally have low cure rates with a prolonged therapy period, a Cochrane review confirmed that high‐quality evidence supports the effectiveness of efinaconazole for the complete cure of tinea unguium. Combination therapy is a way to improve the cure rate of onychomycosis. In this study, topical efinaconazole was administrated to 12 patients who had been treated with oral terbinafine (125 mg daily) for more than 20 weeks with little expected effect. Because terbinafine accumulates for a long time in the nail, treatment immediately followed by other drugs can be considered sequential combination therapy. During terbinafine monotherapy, the percentage involvement decreased from 53.5% to 44.0% after 37.4 weeks and the effective and cure rates were 16.7% and 0%, respectively. During sequential topical efinaconazole therapy combined with lasting terbinafine in the nail, the percentage involvement decreased from 44.0% to 18.7% after 28.4 weeks, and the effective and cure rates were 66.7% and 16.7%, respectively. The improvement rate per month of combination therapy (12.6%) was higher than that with monotherapy (2.1%) (*p* = 0.002). There were no serious side‐effects. This sequential combination therapy with efinaconazole was effective in poor terbinafine responders, making it a promising regimen for improving the cure rate of tinea unguium.

## 1 INTRODUCTION

Efinaconazole is a topical antifungal drug used to treat tinea unguium that was approved by the Japanese government in 2014. A phase 3 study enrolled patients with a nail involvement ratio of 20–50% (mean, 36.7%), and the complete cure rate at 52 weeks was 17.8% (117/656).^1^ Furthermore, a multicenter study including severe cases with a mean nail involvement of 49.1% showed that continuous application up to 72 weeks contributed to a relatively high cure rate of 31.1% (68/223).^2^ A recent Cochrane review of treatments for onychomycosis confirmed that high‐quality evidence supports the effectiveness of efinaconazole for achieving a complete cure.^3^ Oral terbinafine at 250 mg daily for 12 weeks and oral itraconazole pulse therapy (3 cycles of 400 mg daily) are mainly applied internationally, with complete cure rates of 35% and 14%, respectively.^4^ Combination therapy can be administrated in parallel or sequentially. Parallel therapy is recommended for patients who are not likely to respond to the therapy (e.g., patients with diabetes), whereas sequential therapy is recommended for patients who have shown a poor response to initial treatment.^5^ Combination therapy is one way of improving the cure rate of onychomycosis. We herein report the efficacy of topical efinaconazole in sequential combination therapy with oral terbinafine.

## 2 METHODS

This retrospective, single‐center, case series study was approved by the research ethics committee of Noguchi Dermatology Clinic (approval no. 24).

Patients with tinea unguium diagnosed by direct microscopy were treated with oral terbinafine (125 mg daily) for at least 20 weeks. After excluding the patients with a high efficacy rate (>70%), efinaconazole 10% solution was commenced within 1 month. Among all outpatients with onychomycosis between August 2015 and December 2020, 38 were treated with topical efinaconazole as sequential combination therapy. After excluding 15 patients who never visited and 11 patients whose records were lacking imaging data from after the application of efinaconazole, 12 patients (male, n = 6; female, n = 6; mean age ± standard deviation, 65.4 ± 14.7 years) were analyzed. Of note, one limitation of this retrospective study is that the high dropout rate may have caused a selection bias.

For each patient, one of the big toenails was selected as the target nail. The clinical picture of the target nail was evaluated at every visit, and the percentage involvement (affected nail area relative to the entire nail area) was calculated using the image analysis software program Image J 1.44p (National Institutes of Health, Bethesda, MD, USA).

The therapeutic regimen comprised oral terbinafine monotherapy and subsequent consolidation with efinaconazole. Because the therapeutic concentration of terbinafine in the nails lasts for at least 30 weeks after the completion of treatment,^6^ the sequential topical efinaconazole application was considered combination therapy.

Treatment progress (efficacy) was rated on a 5‐point scale as cured, markedly improved, improved, slightly improved, or no change based on the following definitions: (i) “cured” was the complete replacement of the affected nail with a healthy nail; (ii) “markedly improved” was the replacement of the affected nail with more than 70% healthy nail; (iii) “improved” was the replacement of the affected nail with 40–70% healthy nail; (iv) “slightly improved” was the replacement of the affected nail with less than 40% healthy nail; and (v) “no change” was no change or an increase in the affected nail area.

A paired *t*‐test was used to compare the improvement rate (i.e., decrease in the percentage involvement) between groups.

## 3 RESULTS

**FIGURE 1:**
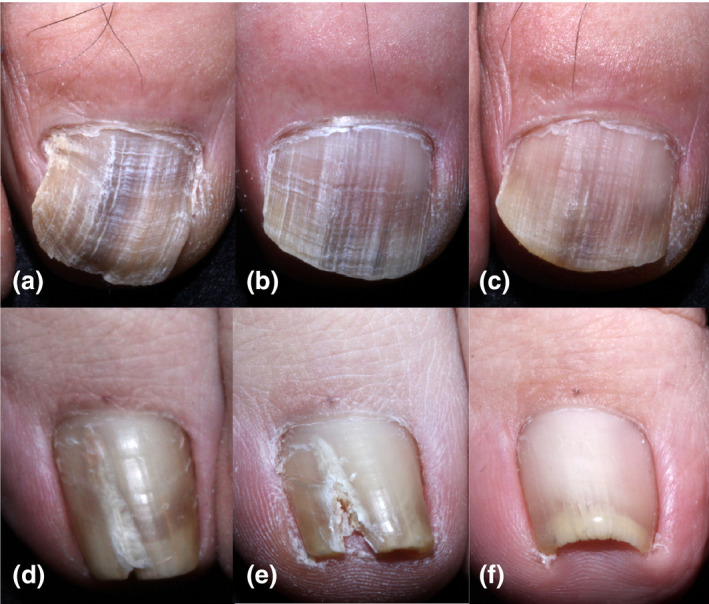
(a) A 61‐year‐old man presented with distal and lateral subungual onychomycosis in the right big toenail. The percentage involvement of his nail was 100%. (b) Oral terbinafine treatment decreased the percentage involvement area to 73.0% after 68 weeks. The improvement rate was 27.0%. (c) Topical efinaconazole treatment decreased the percentage involvement to 55.0% after 61 weeks. The improvement rate per month was 24.7%. The efficacy was considered “slightly improved”. (d) A 37‐year‐old woman presented with onychomycosis showing a white longitudinal streak on the left big toenail. The percentage involvement was 17.4%. (e) Oral terbinafine treatment increased the percentage involvement area to 20.2% after 20 weeks. The improvement rate was −16.1%. (f) Topical efinaconazole treatment decreased the percentage involvement to 0% after 21 weeks. The improvement rate was 100%. The efficacy was evaluated as “cured”

During terbinafine monotherapy, the percentage involvement decreased from 53.5 ± 30.8% to 44.0 ±25.4% after 37.4 ± 15.5 weeks and the effective (i.e., the percentage of those rated as “improved”, “markedly improved”, and “cured”) and cure rates were 16.7% (2/12) and 0% (0/12), respectively. The improvement rate per month was 2.1% ± 3.5%. After terbinafine therapy, all 12 patients showed a positive KOH test result. During the combination therapy with topical efinaconazole, the percentage involvement decreased from 44.0 ± 25.4% to 18.7 ± 18.2% after 28.4 ± 17.8 weeks and the effective and cure rates were 66.7% (8/12) and 16.7% (2/12), respectively. Thus, the combination therapy was more effective than the monotherapy (Figure 1). The improvement rate per month was 12.6% ±8.3%.

**FIGURE 2:**
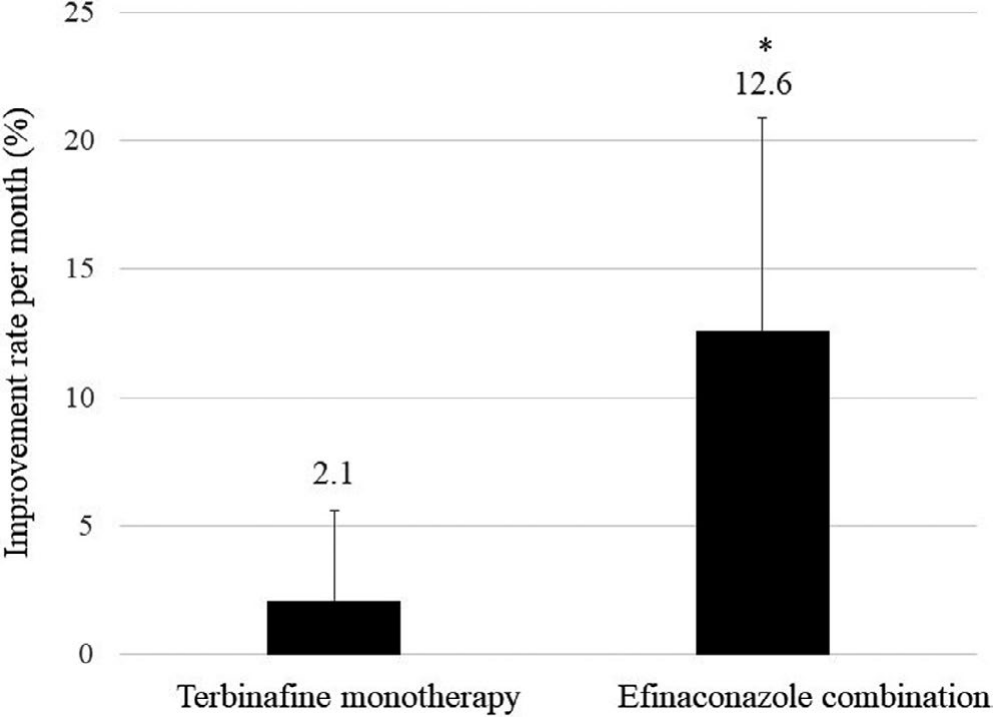
Comparison of the improvement rate between terbinafine monotherapy and sequent combination therapy with efinaconazole. *p = 0.002

The improvement rate with combination therapy was higher than that with monotherapy (*p* = 0.002) (Figure 2). There were no serious side‐effects (Table 1).

**TABLE 1:** Treatment results: efinaconazole subsequently treated with oral terbinafine

Therapy	Duration of therapy (weeks)	Percentage involvement		Improvement rate/month	Evaluation of efficacy					
		Before therapy	After therapy		Cured	Marked improved	Improved	Slightly improved	No change	Effective rate (%)
Terbinafine	37.4 ± 15.5	53.5 ± 30.8	44.0 ± 25.4	2.1 ± 3.5	0	0	2	6	4	16.7
Efinaconazole	28.4 ± 17.8	44.0 ± 25.4	18.7 ± 18.2	12.6 ± 8.3	2	4	2	4	0	66.7

Note

A total of 12 patients were initially treated with terbinafine monotherapy and subsequently treated with efinaconazole.

## 4 DISCUSSION

Terbinafine is the most commonly prescribed oral antifungal medicine approved to treat tinea unguium. The recommended regimen for toenail tinea unguium is 125 mg daily for 24 weeks in Japan.^7^ In daily clinical practice, oral antifungal drugs are effective in many cases; however, in 5.6% (14/247) of cases in our clinic, onychomycosis did not respond to oral terbinafine therapy during the 2‐year study period from 2016 to 2017. One reason for this might be the presence of terbinafine‐resistant dermatophytes. In 2019, we presented a case of tinea unguium caused by a terbinafine‐resistant *Trichophyton rubrum*. Terbinafine is an allylamine derivative that inhibits the enzyme squalene epoxidase (SQLE), thus inhibiting functional fungal cell membrane development. The mutated *SQLE* alleles led to Phe397Leu substitution in the *T. rubrum* SQLE proteins.^8^ Our clinic obtained three terbinafine‐resistant *T. rubrum* strains (3/95, 3.2%, Leu393Phe substitution) in June 2020.

Although onychomycosis caused by non‐dermatophytes occurs at varying frequencies according to the geographic location, in developed countries, approximately 10% of onychomycoses are caused by non‐dermatophyte moulds.^9^, ^10^ We identified 13 (0.5%) cases of non‐dermatophyte onychomycosis among 2591 onychomycoses in 106703 outpatients over the past 5 years (January 2015–December 2019). The pathogens were *Aspergillus* species, *Fusarium* species, *Scopulariopsis* *brevicaulis*, and *Botryosphaeria dothidea*, which is closely related phylogenetically to *Neoscytalidium* *dimidiatum*.^11^ Non‐dermatophyte moulds showed a poor response to terbinafine.^10^, ^11^

Efinaconazole 10% solution is recommended for patients with mild to moderate tinea unguium and assigned a grade of B in Japan.^7^ Efinaconazole is a triazole derivative that inhibits lanosterol 14α‐demethylase and has a low potential to induce drug resistance in dermatophytes.^12^ Furthermore, it shows a broad spectrum of antifungal activities *in vitro* and used to treat patients with non‐dermatophyte mould onychomycosis caused by *Aspergillus* species, *Fusarium* species, and *S. brevicaulis*.^11^

In the present study, the terbinafine concentration in the nails lasted throughout subsequent topical efinaconazole therapy.^6^ Therefore, we presumed that both efinaconazole and terbinafine in the nail worked cooperatively to induce increased antifungal activity. An antifungal susceptibility study *in vitro* using the fractional inhibitory concentration index suggested that terbinafine and efinaconazole had synergic or additive effects on most *T. rubrum* and *Trichophyton* *interdigitale* strains investigated.^13^ The mechanism underlying the synergy effect may involve the blockage of both SQLE and 14α‐demethylase. These *in vitro* synergy effects led to favorable clinical efficacy in the patient’s nail during our sequential combination therapy.

A patient with refractory dermatophytoma to oral terbinafine successfully treated with topical efinaconazole was reported.^14^ The patient applied the medication to the surface of the nail as well as to the hyponychium. Topical efinaconazole targets the fungus from the outside, penetrating the dorsal nail plate, while oral terbinafine works from the inside, penetrating the ventral nail plate. The route of drug delivery to the target site naturally influences the efficacy. Dermatophytoma has been described clinically as a discolored linear band or streak. Not terbinafine but efinaconazole was effective in our case with a longitudinal streak (Figure 1d).

As with our results, topical amorolfine in combination with oral terbinafine was reported to enhance clinical efficacy in comparison to terbinafine alone (59.2% vs. 45.0%).^15^ Although it is difficult under the current Japanese insurance system, parallel combination therapy may be necessary to shorten the treatment period and reduce the dropout rate. Sequential combination therapy with efinaconazole is effective for poor responders to terbinafine and may help improve the cure rate of onychomycosis.

## CONFLICT OF INTEREST

None declared.
